# The oceanic origin of path-independent carbon budgets

**DOI:** 10.1038/s41598-017-10557-x

**Published:** 2017-09-04

**Authors:** Andrew H. MacDougall

**Affiliations:** 0000 0004 1936 7363 grid.264060.6 St. Francis Xavier University, Department of Earth Sciences, Antigonish, B2G 2W5 Canada

## Abstract

Virtually all Earth system models (ESM) show a near proportional relationship between cumulative emissions of CO_2_ and change in global mean temperature, a relationship which is independent of the emissions pathway taken to reach a cumulative emissions total. The relationship, which has been named the Transient Climate Response to Cumulative CO_2_ Emissions (TCRE), gives rise to the concept of a ‘carbon budget’. That is, a finite amount of carbon that can be burnt whilst remaining below some chosen global temperature change threshold, such as the 2.0 °C target set by the Paris Agreement. Here we show that the path-independence of TCRE arises from the partitioning ratio of anthropogenic carbon between the ocean and the atmosphere being almost the same as the partitioning ratio of enhanced radiative forcing between the ocean and space. That these ratios are so close in value is a coincidence unique to CO_2_. The simple model used here is underlain by many assumptions and simplifications but does reproduce key aspects of the climate system relevant to the path-independence of carbon budgets. Our results place TCRE and carbon budgets on firm physical foundations and therefore help validate the use of these metrics for climate policy.

## Introduction

Simulations with Earth system models consistently show a near-linear relationship between cumulative emissions of CO_2_ and change in global mean temperature^1–3^, a relationship which has been shown to be consistent with observations^3^. The relationship is fundamental to the concept of a carbon budget, a finite quantity of cumulative CO_2_ emissions consistent with remaining below a chosen temperature change threshold^4^, such as the 1.5 and 2 °C targets set by the Paris Agreement^5^. Carbon budgets have in turn been used to estimate the fraction of known fossil fuel reserves that can be safely burnt^6, 7^ and to attribute historical responsibility for climate change^8^. Thus carbon budgets have proven a useful tool to conceptualize the challenges inherent in economic decarbonization^9^.

The relationship between cumulative emission of CO_2_ and change in global mean temperature, the slope of which is the Transient Climate Response to Cumulative CO_2_ Emissions (TCRE), has two key features: (1) the relationship is close to linear, that is TCRE is nearly constant; and (2) the relationship is independent of the emissions pathway followed to get to a given quantity of cumulative CO_2_ emissions^1, 10^. Previous efforts to explain the physical origin of TCRE have largely focused on why the relationship is linear^11–13^. However, the second feature of TCRE, its path-independence, is arguably more important for climate policy as one can compute carbon budgets for non-linear temperature versus cumulative emission curves but only if the relationship remains path-independent. The physical origin of the path-independence of carbon budgets has not been fully explained^14^, with only a single study examining the topic^15^.

The near path-independence of carbon budgets is evident in the full spectrum of Earth System Models (ESMs) from simple box models^11, 15^, to intermediate complexity climate models^10, 16^ to full ESMs^17^. Therefore we hypothesize that the path-independence of carbon budgets must arise from some fundamental aspect of carbon cycle dynamics. To explore this hypothesis we will derive a simple model of the Earth system that exhibits path-independent carbon budgets and examine why the model exhibits path-independence. Thus by stripping an Earth system model down to its most fundamental components we hope to derive a model which can be more easily understood.

## Results

### The link between carbon-cycle dynamics and path independence

**Figure 1:**
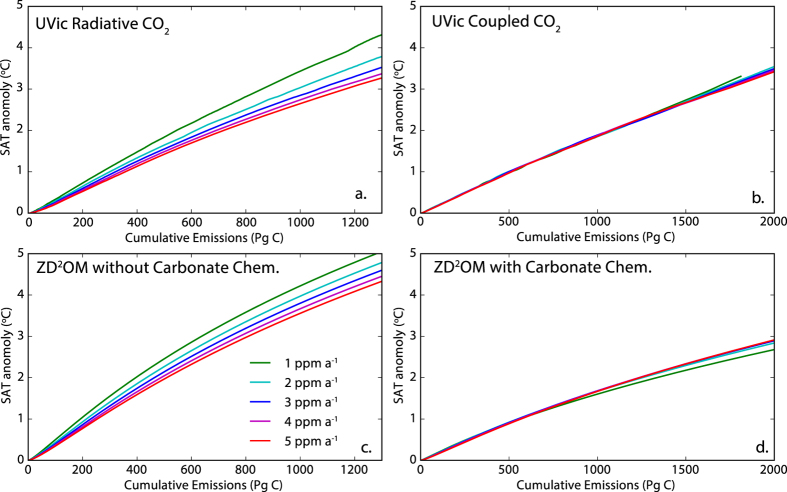
Surface air temperature (SAT) anomaly versus cumulative emissions of CO_2_ curves for: (**a**) simulations with the UVic ESCM forced with CO_2_ that is uncoupled from the model’s biogeochemical components (radiatively coupled CO_2_ – R-CO_2_). (**b**) Simulations with the UVic ESCM forced with fully coupled CO_2_. (**c**) Solutions for the ZD^2^OM not accounting for ocean carbonate chemistry or land carbon uptake. (**d**) Solutions for the full ZD^2^OM. All models are forced with scenarios where atmospheric CO_2_ concentration changes at a constant rate. Note that the horizontal scale is different in the right and left columns as R-CO_2_ emission have an airborne fraction of 1.

The first step in our analysis is to confirm that the path-independence of temperature versus cumulative emission curves arises from modulation of CO_2_ concentration by the simulated carbon cycle. We do this using model simulations with the University of Victoria Earth System Climate Model (UVic ESCM) (see methods) forced with CO_2_ that is decoupled from the model’s biogeochemical components (see Methods) (radiatively coupled – R-CO_2_)^18^. Experiments using radiatively coupled CO_2_ are part of the standard experiment-set conducted for carbon cycle model intercomparison projects^18^. Figure 1a,b shows temperature versus cumulative emission curves for two idealized climate model simulations, one experiment forced with fully coupled CO_2_ and the second experiment forced with R-CO_2_. The figure shows that the temperature versus cumulative emissions curves are nearly linear for R-CO_2_ but with large variation in slope of the curves for simulations with different rates of change of atmospheric R-CO_2_. Consequently the carbon budget for 2 °C of warming for R-CO_2_ increases by 17% when comparing an atmospheric CO_2_ change rate of 2 ppm a^−1^ to a change rate of 5 ppm a^−1^. The same metric for fully coupled CO_2_ changes by just 0.3%. Thus some aspect of the of carbon biogeochemistry is generating the near path-independence of carbon budgets.

### A simple Earth system model

The simple Earth system model devised here is an extension of the analytical approximation of TCRE developed by ref. 13. The function for TCRE derived by ref. 13 was intended to explore why the temperature versus cumulative CO_2_ emission curve is nearly linear, and thus why TCRE was near-constant with respect to cumulative CO_2_ emissions. The paper suggested that the diminishing efficiency of ocean heat uptake and the logarithmic relationship between CO_2_ concentration and radiative forcing compensate in such a way to create the near-constant TCRE with respect to changes in cumulative CO_2_ emissions. The relationship derived by ref. 13 also implied that for path-independence to exist the airborne fraction of carbon must be being modulated as a function of the rate of CO_2_ emissions. Such modulation is evident from the output of Earth System Models^14^ and is believed to originates from ocean carbon uptake^13^. However, the physical mechanism underlying this modulation has remained poorly explained.

For this manuscript we have been able to incorporate a representation of ocean carbonate chemistry into the model using the carbonate-alkalinity approximation of ref. 19, opening the way to finding the physical origin of TCRE path-independence. We have named the model the Zero Dimensional Diffusive Ocean heat and carbon uptake Model (ZD^2^OM). The model is based on ref. 1 definition of TCRE, the forcing response equation from ref. 20, and a relationship for cumulative CO_2_ emissions (see Methods). Combining these relationships with an approximation of heat and carbon uptake as diffusion into a half-space, and the ref. 19 analytical approximation of ocean carbonate chemistry, we find an analytically solvable model approximating TCRE (see Methods). The ZD^2^OM has analytical solutions for linear and exponential growth in atmospheric CO_2_ concentration. Linear pathway solutions for the ZD^2^OM are shown in Fig. 1d and are compared with simulations with the UVic ESCM forced with the same CO_2_ concentration pathways. Also shown in Fig. 1c are linear pathway solutions for a version of the ZD^2^OM without carbon uptake by the ocean or land surface (see Methods). The figure shows that the full ZD^2^OM is able to capture the path-independence characteristic of the TCRE relationship, and that the modified ZD^2^OM is able to capture the path-dependent characteristic of simulations forced with R-CO_2_. Given the land carbon uptake in the ZD^2^OM is a fixed fraction of emissions the figure strongly implies that ocean carbonate chemistry is somehow generating the path-independence of TCRE.

The ZD^2^OM deviates from linearity at lower cumulative emissions than the UVic ESCM. The deviation from linearity is a common feature of models which possess oceans that are too diffusive relative to ocean models with higher fidelity and to the natural ocean^21^. The ZD^2^OM ocean is purely diffusive and therefore the deviation from linearity is expected. Here we are focused on finding the origin of path-independence of carbon budgets, not linearity in the TCRE relationship which is relatively well explored feature^11–13^.

**Figure 2:**
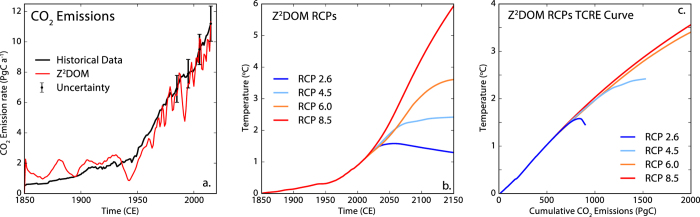
(**a**) Comparison between the ZD^2^OM calculation of CO_2_ emissions rates and historical estimates of anthropogenic CO_2_ emissions rate for the industrial period. ZD^2^OM emission rates are computed from numerical integration of Equations (13), and (16) forced with the historical CO_2_ concentrations. Historical emission estimates are from ref. 46 updated to the present using data from ref. 23. Uncertainty bounds are from Table 6.1 of ref. 22 (**b**) ZD^2^OM estimate of global temperature change under the four Representative Concentration Pathway (RCP) scenarios. (**c**) Temperature versus cumulative CO_2_ emissions curves produced by the ZD^2^OM under the four RCPs.

Figure 2a compares the rate of CO_2_ emissions computed by the ZD^2^OM for the historical era to data-based estimates of emission rates from the same era (see Methods). The ZD^2^OM produces an estimate of the CO_2_ emission rate that is generally consistent with the historical record despite the radical simplicity of the model. Notably the ZD^2^OM emission estimate has a higher inter-annual variability relative to the historical data. The source of this high variability in the ZD^2^OM output is likely the assumption that the land-borne fraction of carbon is constant in time, which contrasts with observational evidence that there is strong inter-annual variability in the uptake of carbon by the terrestrial biosphere^22^. The inter-annual variability in the land carbon sink has been linked to coupled ENSO-forest-fire dynamics and other complex feedbacks^23^. As the ZD^2^OM’s equation for emissions conserves mass, changes in the atmospheric CO_2_ change rate are interpreted as changes in emissions rate instead of changes in the land-borne fraction of carbon. Although the ZD^2^OM only has elementary form solutions for a small number of families of atmospheric CO_2_ pathway functions (linear or exponential growth), the equations which underlie the model can be integrated numerically for any CO_2_ pathway. This feature of the model is demonstrated in Fig. 2b and c, which show the change in global temperature and temperature versus cumulative emission curves produced by the ZD^2^OM when forced with the Representative Concentration Pathways (RCPs)^24^. Note that the ZD^2^OM produces relatively standard temperature versus cumulative emissions curves until the rate of CO_2_ slows near the end of each scenario where path-independence is lost.

**Figure 3:**
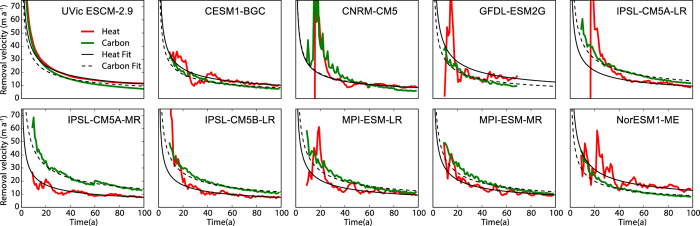
Heat and carbon removal velocities at the ocean surface (see Methods) for nine CMIP5 models and the UVic ESCM. The nine CMIP5 models are those that stored all of the necessary variables to compute removal velocity. Some models stored all the necessary variables to compute heat removal velocity but not carbon removal velocity or vice-versa. See Figures S1 and S2 for all derivable heat and carbon removal velocities respectively. For all models the removal velocity is derived for the idealized 1% yearly change in atmospheric CO_2_ experiment^41^. Black lines show that best fit between the model derived removal velocity and removal velocity computed from the approximation of ocean uptake as diffusion into a half-space. Fits were created by altering the effective ocean diffusivity. If ocean heat and carbon uptake where governed by identical processes in ESMs then the heat and carbon removal velocity functions would be the same.

A critical feature of the ZD^2^OM is that the model treats ocean heat and carbon uptake as diffusion into a half-space. Thus we must check that this assumption approximates heat and carbon uptake in full ESMs. Figure 3 shows the evolution of removal velocity (surface flux divided by the surface concentration anomaly of the fluxed quantity, see Methods) of heat and carbon computed from ESM output from the UVic ESCM and nine models from the Coupled Model Intercomparison Project phase 5 (CMIP5). Using removal velocity facilitates easier comparison of ocean heat and carbon uptake by examining a metric that has the same units for both quantities. If heat and carbon uptake by the ocean were governed by the exact same mechanism in ESMs then the heat and carbon removal velocity functions would be identical. For the models shown in Fig. 3 the velocities for heat and carbon are similar but not identical, consistent with spatial differences in uptake explored by ref. 25 and the biogeochemical processes that affect ocean carbon uptake^22^ but not heat uptake. The figure also shows the fit between the removal velocities and the diffusive approximation of this quantity used to define the ZD^2^OM for heat and carbon, and for each ESM. The fits are generally close after the first 20 years of the simulations (see Supplementary Tables S2 and S3). The figure thus demonstrates that at the global scale ocean heat and carbon uptake in ESMs can be approximated well by diffusion, at least while carbon emissions continue. The values of the ocean diffusion parameter computed from fitting the diffusive approximation to the heat and carbon removal velocities are of a similar order of magnitude, but generally larger in value (0.20 to 8.48 cm^2^s^−1^, median 3.73 cm^2^s^−1^) than the enhanced ocean diffusion value of ~1 cm^2^s^−1^ needed to balance deep water formation, computed originally by ref. 26 (see Supplementary Tables S2 and S3). Enhanced ocean diffusivity schemes are commonly used to close meridional overturning circulation in global ocean models e.g. ref. 27, despite the mechanism having been shown to be very weak in the natural ocean^27^.

A simplified representation of the ZD^2^OM is:

1 $${\rm{\Lambda }}=\frac{R\mathrm{(1}-l)\,\mathrm{ln}(\frac{{C}_{A}}{{C}_{Ao}})}{\lambda }{({\rm{\Delta }}{C}_{A}+{\rm{\Delta }}{C}_{O}+\frac{\kappa }{\lambda }{\rm{\Delta }}{C}_{A}+\frac{\kappa }{\lambda }{\rm{\Delta }}{C}_{O})}^{-1},$$

where Λ is TCRE, *C*_*A*_ is the size of the atmospheric carbon pool, *C*_*Ao*_ is the original size of the atmospheric carbon pool, *C*_*O*_ is the size of the oceanic carbon pool, *κ* is the ocean heat uptake parameter, *λ* is the climate feedback parameter, *R* is radiative forcing from an e-fold increase in atmospheric CO_2_, and *l* is the land-borne fraction of carbon. All variables and constants are defined in in Table 1.

**Table 1:** Variables and constants used in this manuscript.

Variable	Description	Units
*b*	Change rate of atm. CO_2_	PgC a^−1^
*C*_*A*_	Atmospheric carbon pool	PgC
*C*_*O*_	Cumulative ocean carbon uptake	PgC
*D*_*s*_	Ocean suface DIC	mol m^−3^
*E*	Cumulative CO_2_ emissions	PgC
*F*	Radiative forcing	W m^−2^
*L*	Radiative response	W m^−2^
*N*	Ocean/planetary heat uptake	W m^−2^
*T*	Change in global temperature	K
*T*_*o*_	Change sea surface temperature	K
*t*	Time	a
*q*_*o*_	Ocean carbon uptake	PgC a^−1^
*V*_*q*_	Removal velocity	m a^−1^
*β*	Change rate of atm. CO_2_	% a^−1^
*κ*	Ocean heat uptake efficiency	W m^−1^ K^−1^
Λ	TCRE	K EgC^−1^
**Constant**		
*A*_*c*_	Carbonate alkalinity: [HCO_3_^−^] + 2[CO_3_^2−^]	mol m^−3^
*B*_*o*_	Unit conversion constant (carbon)	m^2^ Pg mol^−1^
*C*_*Ao*_	Pre-Industrial atm. CO_2_	PgC
*D*_*so*_	Pre-Industrial ocean suface DIC	mol m^−3^
*f*_*o*_	Planetary ocean fraction	—
*R*	e-fold radiative forcing from CO_2_	W m^−2^
*K*_1_	Equilibrium constant: [HCO_3_^−^][H+]/[CO_2_*]	mol m^−3^
*K*_2_	Equilibrium constant: [CO_3_^2−^][H+]/[HCO_3_^−^]	mol m^−3^
*K*_*o*_	Solubility of CO_2_	ppm^−1^ m^−3^ mol
*l*	Land-borne fraction of carbon	—
*M*_*r*_	CO_2_ atmospheric mixing ratio	PgC ppm^−1^
*α*	Ocean diffusivity	cm^2^s^−1^
Γ	Surface DIC change from e-fold change in atm. CO_2_	mol m^−3^
*ε*	Ratio of ocean to global temperature change	—
*λ*	Climate feedback parameter	W m^−1^K^−1^
*ρC*_*p*_	Heat capacity of water	J m^−3^K^−1^
*τ*	Unit conversion constant (heat)	s a^−1^

Values of the constants used in this manuscript are included in the Supplementary information Table S4. The abbreviation ‘atm.’ stands for atmosphere.

The Δ*C*_*O*_ term in Equation (1) is computed from an integral which includes the evolution of ocean surface Dissolved Inorganic Carbon (DIC) in time (see Methods). If ocean surface DIC evolution is approximated as a logarithmic function of atmospheric CO_2_ concentration then the integrand Δ*C*_*O*_ has elementary form function solutions for linear and exponential atmospheric CO_2_ concentration pathways (see Methods). Here we are interested in why TCRE does not change much given different pathways to get to the same atmospheric CO_2_ concentration. To examine this question within the context of linear changes in atmospheric CO_2_ concentration we select a value of atmospheric CO_2_ and explore the effect of rate of atmospheric CO_2_ change, *b*, on the TCRE relationship. A mathematically convenient value of atmospheric CO_2_ concentration is double the pre-industrial CO_2_ concentration (*C*_*A*_ = 2 × *C*_*Ao*_), which gives the relationship:

2 $${\rm{\Lambda }}=\frac{R\mathrm{(1}-l)\,\,\mathrm{ln}\,\mathrm{(2)}}{\lambda }{\times ({C}_{Ao}+\frac{2{B}_{o}{\rm{\Gamma }}\sqrt{{C}_{Ao}}}{\sqrt{b\mu }}(\frac{\pi }{2}+\mathrm{ln}\mathrm{(2)}-2)+\frac{{f}_{o}\rho {c}_{p}\tau \varepsilon \sqrt{b{C}_{Ao}}}{\lambda \sqrt{\mu }}+\frac{2{f}_{o}\rho {c}_{p}\tau {B}_{o}{\rm{\Gamma }}}{\mu \lambda }(\frac{\pi }{2}+\mathrm{ln}\mathrm{(2)}-2))}^{-1},$$

where *B*_*o*_ is a unit conversion constant, Γ change in ocean surface DIC from an e-fold increase in atmospheric CO_2_, *μ* is constant related to ocean diffusivity (see Methods), *f*_*o*_ is the fraction of the Earth covered by oceans, *ρc*_*p*_ is the heat capacity of water, *τ* is the conversion factor between seconds and years, *ε* is the ratio of sea surface to global surface temperature change. Two of the terms in the denominator of the Equation (2) are functions of *b*. Thus if TCRE is constant for different values of *b* then a change in *b* must cause an increase in the value of one term and a decrease in the value of the other term that cancel out almost exactly. Looking back to Equation (1) the two terms which contain *b* are equivalent to:

3 $$\frac{2{B}_{o}{\rm{\Gamma }}\sqrt{{C}_{Ao}}}{\sqrt{b\mu }}(\frac{\pi }{2}+\,\mathrm{ln}\,\mathrm{(2)}-2)={\rm{\Delta }}{C}_{O},and$$

4 $$\frac{{f}_{o}\rho {c}_{p}\tau \varepsilon \sqrt{b{C}_{Ao}}}{\lambda \sqrt{\mu }}=\frac{\kappa }{\lambda }{\rm{\Delta }}{C}_{A},$$

**Figure 4:**
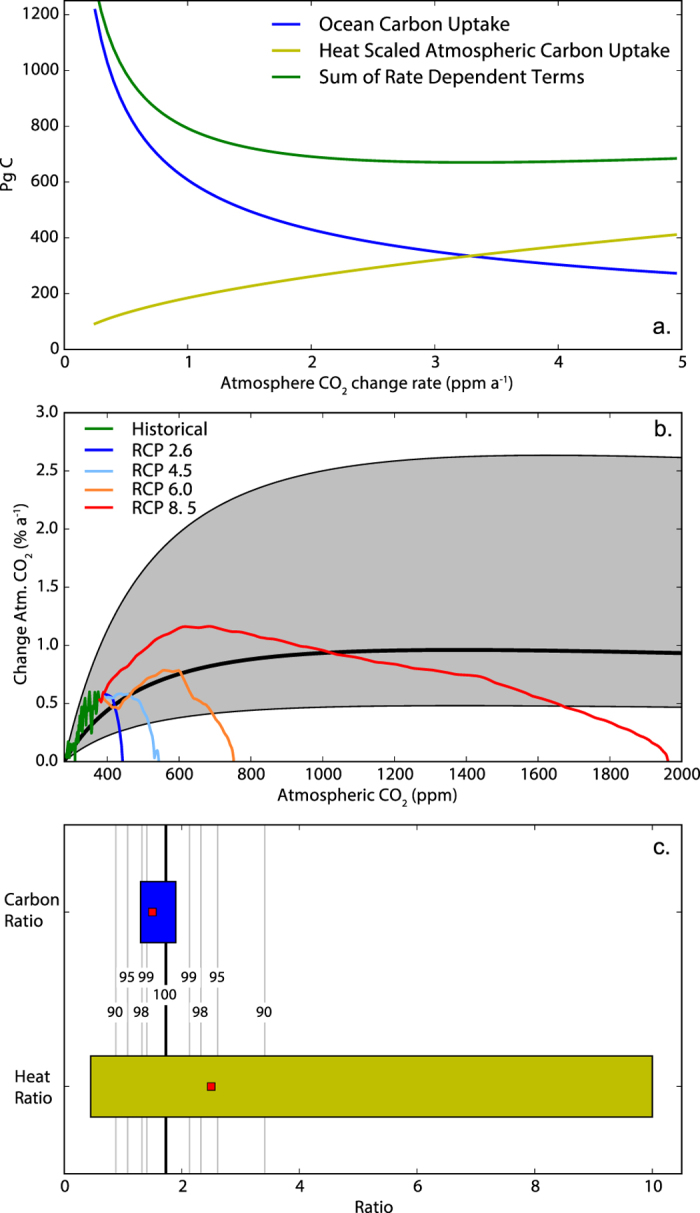
(**a**) Rate dependent terms from Equation (1) evaluated at the time of doubled atmospheric CO_2_. Terms are a function of the rate of CO_2_ change *b* At high atmospheric CO_2_ change rates cumulative ocean carbon uptake is lower while ocean heat uptake efficiency (*κ*) is higher. These two factors compensate exactly where the two terms have the same value and nearly compensate over a wide range of rates of atmospheric CO_2_ change centred about the point of exact cancelation. (**b**) Grey envelope encompasses region where the ZD^2^OM predicts TCRE should be nearly path-independent, defined as where change in the rate dependent terms compensate by greater than 95%. Thick black line is the atmospheric CO_2_ change rate at which the terms cancel exactly. Plotted atop this envelope are the historical rates of atmospheric CO_2_ change and the rates from the four RCP scenarios. (**c**) Fifth to ninety-fifth percentile ranges for the carbon and heat ratios as calculated from observation derived parameters. Red square is the median estimate for each. Black line is the value of the ratios that the ZD^2^OM predicts exact rate compensation would occur. Grey lines are labeled by the fraction of rate compensation that is predicted to occur with a heat ratio at that value.

Figure 4a shows the evolution of these terms as a function of atmospheric CO_2_ change rate (*b*). That is, the x-axis represents different CO_2_ pathways. As *b* increases the ocean has less time to absorb carbon such that C_*O*_ is smaller than it would be for a pathway that takes more time to reach the same atmospheric CO_2_ concentration. Simultaneously the increase in *b* makes ocean heat uptake more efficient and thus *κ* larger. These effects nearly cancel to create stable value for the denominator of Equation (1), and thus a path-independent value of TCRE. The derivatives of the two terms cancel exactly where the two terms are of equal value and nearly cancel for a broad range of *b* values near the point of exact cancelation. This cancelation feature is a mathematical consequence of the one term being a function of $\sqrt{b}$ and the other being a function of $\frac{1}{\sqrt{b}}$ (see Supplementary information for a formal proof). This mathematical form arises in the ZD^2^OM from the ocean heat and carbon uptake both being driven by diffusion. In addition one quantity must be instantaneous (ocean heat uptake) and thus take on the inverse square root form, and the other quantity must be an time integral quantity (cumulative ocean carbon uptake) and thus take-on the square root function from, for the cancellation effect to emerge.

The point where the two rate-dependent terms are equal is where their derivatives are of equal magnitude but of opposite sign and this point is therefore at the core of the pathway-independence of TCRE. By examining the exact cancelation point we can illuminate physically why the terms are close in value under ambient conditions. Setting the terms equal and collecting all of the carbon parameters to one side and the heat parameters to the other we find:

5 $$\frac{{\rm{\Delta }}{C}_{A}}{{\rm{\Delta }}{C}_{O}}=\frac{L}{N}$$

where *L* is the enhanced longwave radiation to space (radiative response) from the Earth’s surface (*L* = *λT*), and *N* is ocean heat uptake (*N* = *κT*). Thus, these equations state that for a certain rate of atmospheric CO_2_ change the cumulative partitioning of anthropogenic carbon between the ocean and the atmosphere is the same as the instantaneous partitioning of anthropogenically enhanced radiative forcing between the ocean and space. The equivalency depends on the particular values for the parameters that govern ocean carbonate chemistry (summarized in Γ). As far as we can discern there is no fundamental reason that these ratios are so close in value, and no equivalent relationship is expected for non-CO_2_ greenhouse gases at their present day emission rates. For example N_2_O and CH_4_ are both held largely in the atmosphere, with atmospheric fractions at ~80%^22, 28^ and 99%^29^ of the atmosphere-ocean reservoir respectively. Therefore ocean uptake of these gases is expected to be small^22^ and the equivalent of the left-hand ratio of Equation (5) should be much larger than the right-hand ratio for these gases (the right-hand side is independent of radiative forcing agent). However, very slow emissions of N_2_O may allow a match between the Δ*N*_2_ *O*_*atm*_/Δ*N*_2_ *O*_*ocean*_ ratio and the *L*/*N* ratio.

Figure 4b shows the envelope where TCRE should be within 95% of its theoretical peak value, as a function of atmospheric CO_2_ concentration and rate of atmospheric CO_2_ change. Plotted on top of this envelope are the pathways of historical CO_2_ change and the four RCP scenarios used in the last Intergovernmental Panel on Climate Change (IPCC) assessment report^30^. The figure shows that the historical CO_2_ pathway falls within the envelope expected to maintain path-independence of TCRE. The RCP scenarios fall within the envelope until atmospheric CO_2_ concentration is stabilized near the end of each scenario. Therefore the ZD^2^OM predicts that carbon budgets should be near path-independent for all plausible future emission scenarios.

### Observation based estimates of the heat and carbon ratios

Values for the ‘heat ratio’ $(\frac{L}{N})$ and the ‘carbon ratio’ $(\frac{{\rm{\Delta }}{C}_{A}}{{\rm{\Delta }}{C}_{O}})$ can be computed from observation based values of atmospheric CO_2_ change, ocean carbon uptake, total radiative forcing, and ocean heat uptake (see Methods). These observation based estimates give a contemporary value of the carbon ratio of 1.5 [1.3 to 1.9] and a value for the heat ratio of 2.5 [0.45 to 10] where the values in brackets are the 5th to 95th percentile range. Figure 4c shows the range of these observation based estimates in addition to the theoretical bounds where the ratios should be close to path-independent, derived from Equation (15). The figure shows that the central estimates of the heat and carbon ratios are within the bounds that should create near path-independence. However, the large uncertainty associated with radiative forcing^31^ and ocean heat uptake^32^ mean that we cannot exclude the possibility that the ratios in the natural world are beyond the bounds that would create path-independence TCRE and carbon budgets.

## Discussion

Our analysis suggests that one of the key features that generates the path-independence of TCRE in the ZD^2^OM is that heat and carbon uptake by the ocean are approximated by diffusion. However, simple ESMs and intermediate complexity ESMs generally have more diffusive oceans than full ESMs^21^. Additionally, because full ESMs are less diffusive they exhibit more linear temperature versus cumulative emission curves^33^. Thus the two properties that make carbon budgets useful for climate policy, linearity and path-independence, may play off one-another. More diffusive models are less linear and more path-independent, and less diffusive models are more linear and less path-independent. Such an effect may explain the path-dependent temperature versus cumulative emission curves shown by ref. 34 generated by the not very diffusive GFDL-ESMG2 (see Fig. 3). Full ESMs are likely to better represent the natural world than intermediate complexity models and thus carbon budgets for policy purposes may have to account for some path-dependence.

The study of ref. 15 used a similar approach to the approach used here to examine the origin of path-independence in temperature versus cumulative emission curves. The study examined a linear two-box ocean model and showed that for path-independence to exist the timescale for increase in cumulative emissions must be much greater than the timescale for decrease in airborne fraction of CO_2_ and much smaller than the damping-timescale of the ocean^15^. Thus path-independence should exist for all but very slow emission rates, consistent with the lower and upper bounds of the shaded area from Fig. 4b. The study of ref. 15 implicitly accounts for carbon biogeochemistry using impulse-response functions derived form ESM simulations^35^. Thus the study was unable to explicitly account for the role in ocean carbonate chemistry in generating path-independence.

The relationship between cumulative emissions of CO_2_ and change in global temperature has three features that make derivation of carbon budgets useful. (1) The relationship is nearly linear, thus a gram of carbon emitted 100 years ago has the same impact on climate as a gram of carbon emitted today or 100 years from now. (2) The relationship is nearly path-independent such that the pathway followed to get to an emission total is irrelevant. (3) Warming following cessation of CO_2_ emissions is either close to zero^36^, or will peak and stabilize^2, 25^. Here we have provided a simple hypothesis for the existence of the second feature, path-independence. However, the model used here is unsuited for exploring the stability of warming following cessation of emissions. The close-to-zero post-cessation warming intuitively should arise from compensation between ocean heat and carbon uptake^25, 36^ but more work is needed to fully explain the mechanism behind this feature of ESM output.

The analysis presented here suggests that the path-independence of TCRE will breakdown as emissions approach zero (Fig. 4c). As most emission scenarios envision a gradual reduction of emissions to zero^30^ this effect may be important for computing the final value of the carbon budget for a given temperature target. Thus investigating the effect of long-tail low-rate emissions on the carbon budget may be an opportunity for research with more complex Earth system models.

Here we have shown that the path-independence of TCRE and therefore carbon budgets arises from: (1) ocean heat and carbon uptake being governed by a similar mechanism which at the global average is consistent with diffusion into a half-space. (2) The partitioning ratio of anthropogenic carbon between the ocean and the atmosphere being close in value the partitioning ratio of enhanced radiative forcing between the ocean and space. The first factor should apply to any long-lived greenhouse gas that is soluble in sea-water. The second factor, however, is a deep coincidence likely applicable only to CO_2_ and is the core property that generates the path-independence of carbon budgets. The heat and carbon ratios do appear to be close in the natural world, however confirming whether at the global average the natural ocean heat and carbon uptake can be approximated by diffusion, or whether the fit to diffusion is a property of ESMs that is an artifact of using refs 26, 27 based closure schemes in ESMs, is critical for validating the path-independence of carbon budgets.

## Methods

### UVic ESCM

The University of Victoria Earth System Climate model 2.9 (UVic ESCM 2.9) is a climate model of intermediate complexity composed of a full three dimensional ocean general circulation model coupled to a simplified energy and moisture balance atmosphere^37^. The model contains a full realization of the oceanic^38^ and terrestrial carbon cycles^39, 40^. The simulations with the UVic ESCM shown in Fig. 1 were forced with atmospheric CO_2_ concentration pathways where CO_2_ changes at a constant rate of 1 to 5 ppm a^−1^ in intervals of 1 ppm a^−1^. Simulations with radiatively coupled CO_2_ were conducted using the protocol of ref. 18. Output from the UVic ESCM used to compute removal velocities for heat and carbon are from the 1% annual change in atmospheric CO_2_ concentration experiment, a standard benchmarking experiment used for model intercomparison^41^. For all simulations atmospheric CO_2_ concentration is prescribed and emissions of CO_2_ are diagnosed as the residual term of the carbon-cycle mass balance.

### Derivation of the ZD^2^OM

The zero dimensional diffusive ocean model (ZD^2^OM) is a model of energy and carbon uptake by the Earth’s ocean that is simple enough to be amenable to analysis. The model is derived from the ref. 1 definition of the TCRE, the forcing-response equation^20^, the ocean carbonate chemistry approximation of ref. 19, and the assumption that the ocean heat and carbon uptake are governed by diffusion into a half-space. Here we present a summarized derivation of the model. A complete derivation is included in the Supplementary materials.

The ref. 1 definition of TCRE is:

6 $${\rm{\Lambda }}=\frac{T}{E},$$

where Λ is TCRE, *T* is the change in global mean temperature, and *E* is cumulative emissions of CO_2_. For the ZD^2^OM both *T* and *E* are subject to approximations. Global mean temperature change is derived from the forcing-response equation^20^:

7 $$F=\lambda T+N,$$

where *F* is the radiative forcing, *λ* is the climate feedback parameter, and *N* is planetary heat uptake. We assume that all radiative forcing is from changes in the atmospheric concentration of CO_2_ and that all heat uptake goes into the ocean. Thus we approximate *F* using the classical approximation of radiative forcing from CO_2_^42^:

8 $$F=R\,\mathrm{ln}(\frac{{C}_{A}}{{C}_{Ao}}),$$

where *R* is the radiative forcing from an e-fold increase in CO_2_, *C*_*A*_ is the atmospheric CO_2_ content, and *C*_*Ao*_ is the pre-industrial atmospheric CO_2_ content. *N* is approximated as heat diffusion into a half-space:

9 $$\frac{N}{{f}_{o}}=\frac{\rho {C}_{p}\sqrt{\alpha }(1+\sqrt{\pi }){T}_{o}}{\pi \sqrt{t}},$$

where *f*_*o*_ is the fraction of the planet covered by ocean, *ρC*_*p*_ is the volumetric heat capacity of water, *T*_*o*_ is the global mean sea surface temperature anomaly, *τ* is the conversion factor between seconds and years, *α* is the diffusivity of the ocean, and *t* is time. To solve for temperature, *N* is represented by *N* = *κ*(*t*)*T*, where *κ* is the classical ocean heat uptake efficiency e.g. ref. 43, here taken to be a time-evolving function instead of a constant e.g. ref. 13. Substituting the above relation into Equation (9) we find:

10 $$\kappa (t)=\frac{{f}_{o}\rho {C}_{p}\varepsilon \sqrt{\alpha }(1+\sqrt{\pi })}{\pi \sqrt{t}},$$

where *ε* is the ratio of sea surface temperature change to global temperature change, taken to be a constant. Analysis of National Oceanic and Atmospheric Administration (NOAA) ocean and global temperature change products shows that the decadal average values of ε vary between 0.79 to 0.85 since the 1950s without a clear secular trend^44^ (see Table S1). Analysis of the output of climate model simulated sea surface temperature anomalies and 2 m air temperature anomaly output also suggest that *ε* is relatively constant (See Figure S1).

To reduce the number of constants we define $\mu =\frac{{\pi }^{2}}{\alpha {(1+\sqrt{\pi })}^{2}}$ and γ = *f*_*o*_ *ρC*_*p*_ *τε*. Solving for temperature we find:

11 $$T=\frac{R}{\lambda }(\frac{\mathrm{ln}(\frac{{C}_{A}}{{C}_{Ao}})}{1+\frac{\gamma }{\sqrt{\mu {\lambda }^{2}t}}})\mathrm{.}$$

Cumulative emissions are the sum of the carbon added to the atmosphere, ocean, and terrestrial biosphere from anthropogenic sources. For the ZD^2^OM we assume that the uptake by land is a constant fraction of anthropogenic emissions (*l*) and that the change in the atmospheric carbon pool is known. The estimated value of the land-borne fraction of carbon exhibits high uncertainty and strong inter-annual and decadal variability^22^. With no clear pattern in the decadal value of the parameter assuming a constant value in time appears to be the most parsimonious assumption. Therefore the evolution of emissions is given by the equation:

12 $$E=\frac{1}{1-l}({C}_{A}-{C}_{Ao}+{\int }_{o}^{t}{q}_{o}dt),$$

where *q*_*o*_ is the flux of carbon into the ocean. We approximate *q*_*o*_ using a similar approximation as that used for *κ*:

13 $${q}_{o}={B}_{o}\frac{{D}_{s}-{D}_{so}}{\sqrt{\mu t}},$$

where *D*_*s*_ is the surface Dissolved Inorganic Carbon (DIC) of the ocean, *D*_*so*_ is the pre-industrial surface DIC of the ocean and *B*_*o*_ is a conversion factor equal to ${B}_{o}=\frac{{A}_{o}M}{1\times {10}^{15}}$, and *A*_*o*_ is the area of the ocean, *M* is the molar mass of carbon, and 1 × 10^15^ is the conversion factor between g and Pg. Substituting Equation (13) into Equation (12) we find:

14 $$E=\frac{1}{1-l}({C}_{A}-{C}_{Ao}+{\int }_{o}^{t}{B}_{o}\frac{{D}_{s}-{D}_{so}}{\sqrt{\mu t}}dt)\mathrm{.}$$

With functions for temperature and cumulative emissions of CO_2_ we can derive the function for TCRE:

15 $${\rm{\Lambda }}=\frac{R\mathrm{(1}-l)}{\lambda }(\frac{\mathrm{ln}(\frac{{C}_{A}}{{C}_{Ao}})}{1+\frac{\gamma }{\sqrt{\mu {\lambda }^{2}t}}})(\frac{1}{{C}_{A}-{C}_{Ao}+{\int }_{o}^{t}{B}_{o}\frac{{D}_{s}-{D}_{so}}{\sqrt{\mu t}}\,dt})\mathrm{.}$$

To further evaluate Equation (15) we must link the change in atmospheric CO_2_ to the change in the surface concentration of DIC (*C*_*A*_ and *D*_*s*_). To investigate this link we incorporate an approximation of ocean carbonate chemistry into our model.

For our purposes we wish to solve the carbonate system analytically to derive a relationship between atmospheric CO_2_ concentration and ocean surface DIC. To make this possible we will make three simplifying assumptions: 1) we will ignore the temperature and salinity dependence of the carbonate equilibrium constants K_1_, K_2_, and CO_2_ solubility constant K_*o*_. 2). We will approximate alkalinity as carbonate alkalinity following refs 3, 19) we will assume that at the surface the partial pressure of CO_2_ in the ocean is equal to the partial pressure of CO_2_ in the atmosphere. For the full derivation of Equation (16) see the Supplementary Information. From these assumptions we derive the approximation:

16 $${D}_{s}=\frac{1}{2}\sqrt{\frac{{K}_{1}^{2}{K}_{o}^{2}}{16{K}_{2}^{2}{M}_{r}^{2}}{C}_{A}^{2}+\frac{{K}_{1}{K}_{o}}{2{K}_{2}{M}_{r}}{C}_{A}{A}_{c}}+(\frac{{K}_{o}}{{M}_{r}}-\frac{{K}_{1}{K}_{o}}{8{K}_{2}{M}_{r}}){C}_{A}+\frac{{A}_{c}}{2},$$

where *A*_*c*_ is the carbonate alkalinity *A*_*c*_ = [HCO_3_^−^] + 2[CO_3_^2−^] and *M*_*r*_ is the conversion factor between the mass of CO_2_ in the atmosphere and the partial pressure of CO_2_ in the atmosphere. The above equation can be substituted into the equation for TCRE (Equation (15)) to solve to the evolution of TCRE in time. However, the integral in the equation, ${\int }_{o}^{t}{B}_{o}\frac{{D}_{s}-{D}_{so}}{\sqrt{\mu t}}dt$, can only be solved using numerical integration. As we wish to derive as a system that can be analyzed analytically we therefore approximate *D*_*s*_ using a log-space Taylor series approximation of Equation (16) centred about *C*_*A*_ = *C*_*Ao*_:

17 $${D}_{s}\approx ({C}_{Ao}(\frac{{K}_{o}}{{M}_{r}}-\frac{{K}_{1}{K}_{o}}{8{K}_{2}{M}_{r}})+\frac{\frac{{K}_{1}^{2}{K}_{o}^{2}}{8{K}_{2}^{2}{M}_{r}^{2}}{C}_{Ao}^{2}+\frac{{K}_{1}{K}_{o}}{2{K}_{2}{M}_{r}}{C}_{Ao}{A}_{c}}{4\sqrt{\frac{{K}_{1}^{2}{K}_{o}^{2}}{16{K}_{2}^{2}{M}_{r}^{2}}{C}_{Ao}^{2}+\frac{{K}_{1}{K}_{o}}{2{K}_{2}{M}_{r}}{C}_{Ao}{A}_{c}}})\mathrm{ln}(\frac{{C}_{A}}{{C}_{Ao}})+{D}_{so}.$$

This approximation of *D*_*s*_ is within 0.5% accurate of the full function up to an atmospheric CO_2_ concentration of 600 ppm (see Figure S5 in Supplementary information). As a check on the logarithmic approximation of DIC evolution and on the other simplifying assumptions outlined above, the surface DIC anomaly computed from Equation (17) is compared to output from the UVic ESCM in Figure S4. The figure shows that the surface DIC anomaly produced by Equation (17) closely matches that from the model output. At the point of doubled atmospheric CO_2_ concentration Equation (17) underestimates the DIC anomaly by 2.6%. With the *D*_*s*_ approximation in-hand we can derive an analytically amenable function for TCRE.

For simplicity we define a constant Γ to represent the factor in front of the logarithm:

18 $${\rm{\Gamma }}=({C}_{Ao}(\frac{{K}_{o}}{{M}_{r}}-\frac{{K}_{1}{K}_{o}}{8{K}_{2}{M}_{r}})+\frac{\frac{{K}_{1}^{2}{K}_{o}^{2}}{8{K}_{2}^{2}{M}_{r}^{2}}{C}_{Ao}^{2}+\frac{{K}_{1}{K}_{o}}{2{K}_{2}{M}_{r}}{C}_{Ao}{A}_{c}}{4\sqrt{\frac{{K}_{1}^{2}{K}_{o}^{2}}{16{K}_{2}^{2}{M}_{r}^{2}}{C}_{Ao}^{2}+\frac{{K}_{1}{K}_{o}}{2{K}_{2}{M}_{r}}{C}_{Ao}{A}_{c}}}).$$

This factor is equivalent to the increase in ocean surface DIC given an e-fold increase in atmospheric CO_2_ concentration. Substituting this relationship in the equation for ocean carbon flux (Equation (13)) we find:

19 $${q}_{o}=\frac{{B}_{o}{\rm{\Gamma }}\,\mathrm{ln}(\frac{{C}_{A}}{{C}_{A}o})}{\sqrt{\mu t}},$$

and the relationship for TCRE becomes:

20 $${\rm{\Lambda }}=\frac{R\mathrm{(1}-l)}{\lambda }(\frac{\mathrm{ln}(\frac{{C}_{A}}{{C}_{Ao}})}{1+\frac{\gamma }{\sqrt{\mu {\lambda }^{2}t}}})(\frac{1}{{C}_{A}-{C}_{Ao}+{B}_{o}{\rm{\Gamma }}{\int }_{0}^{t}\frac{\mathrm{ln}(\frac{{C}_{A}}{{C}_{Ao}})}{\sqrt{\mu t}}dt})\mathrm{.}$$

The approximate form of the ZD^2^OM shown in Equation (20) can be solved analytically for certain CO_2_ concentration pathways. Two pathways relevant to the climate problem are a linear increase in atmospheric CO_2_ concentration *C*_*A*_ = *bt* + *C*_*Ao*_, and a exponential increase in atmospheric CO_2_ concentration *C*_*A*_ = *C*_*Ao*_ *e* ^*βt*^, where *b* is the rate of change in atmospheric CO_2_ content (Pg a^−1^) and *β* is the growth rate of atmospheric CO_2_ content (% a^−1^). The linear solution is:

21 $$\begin{array}{rcl}{\rm{\Lambda }} & = & \frac{R\mathrm{(1}-l)}{\lambda }(\frac{\mathrm{ln}(\frac{{C}_{A}}{{C}_{Ao}})}{1+\frac{\gamma \sqrt{b}}{\sqrt{\mu {\lambda }^{2}({C}_{A}-{C}_{Ao})}}})\\  &  & \times (\frac{1}{{C}_{A}-{C}_{Ao}-\frac{4{B}_{o}{\rm{\Gamma }}}{\sqrt{b\mu }}(\sqrt{{C}_{A}-{C}_{Ao}}-\sqrt{{C}_{Ao}}\arctan (\frac{\sqrt{{C}_{A}-{C}_{Ao}}}{\sqrt{{C}_{Ao}}}))+\frac{2{B}_{o}{\rm{\Gamma }}}{\sqrt{b\mu }}\,\mathrm{ln}(\frac{{C}_{A}}{{C}_{Ao}})\sqrt{{C}_{A}-{C}_{Ao}}}),\end{array}$$

and the solution for an exponential increase in atmospheric CO_2_ concentration is:

22 $${\rm{\Lambda }}=\frac{R\mathrm{(1}-l)}{\lambda }(\frac{\mathrm{ln}(\frac{{C}_{A}}{{C}_{Ao}})}{1+\frac{\gamma \sqrt{\beta }}{\sqrt{\mu {\lambda }^{2}\,\mathrm{ln}(\frac{{C}_{A}}{{C}_{Ao}})}}})(\frac{1}{{C}_{A}-{C}_{Ao}+\frac{2{B}_{o}{\rm{\Gamma }}\,\mathrm{ln}\,{(\frac{{C}_{A}}{{C}_{A}o})}^{\frac{3}{2}}}{3\sqrt{\mu \beta }}})\mathrm{.}$$

### ZD^2^OM without carbonate chemistry

To compare the ZD^2^OM with ESM simulations forced with R-CO_2_ a version of the ZD^2^OM with no carbonate chemistry and no uptake of carbon by land was derived. The temperature function is unaffected by carbon uptake and thus remains Equation (11). The function for emissions will simply be:

23 $$E={C}_{A}-{C}_{Ao}\mathrm{.}$$

The equation for TCRE of R-CO_2_ therefore is:

24 $${{\rm{\Lambda }}}_{R-CO2}=\frac{R}{\lambda ({C}_{A}-{C}_{Ao})}(\frac{{\rm{l}}{\rm{n}}(\frac{{C}_{A}}{{C}_{Ao}})}{1+\frac{\gamma }{\sqrt{\mu {\lambda }^{2}t}}})$$

### Removal velocity

The removal velocity describes how efficient the ocean is at redistributing quantities at the ocean surface into the ocean interior. Removal velocity is defined as the ratio of the flux into the ocean over the anomaly of the surface concentration of the quantity being fluxed. That is, if we are given a flux the removal velocity is the instantaneous rate at which the substance must be removed from the surface to maintain the present surface concentration of the substance. The relationship has units of velocity (m*s* ^−1^). The relationship for heat is:

25 $${V}_{q}=\frac{N}{{f}_{o}\rho {C}_{p}{T}_{o}},$$

where *V*_*q*_ is the removal velocity. The relationship for carbon is:

26 $${V}_{q}=\frac{{q}_{o}}{{B}_{o}({D}_{s}-{D}_{so})}\mathrm{.}$$

The diffusive approximation of ocean heat and carbon uptake predicts that the removal velocity to be:

27 $${V}_{q}=\frac{1}{\sqrt{\mu t}}\mathrm{.}$$

Removal velocity can be computed from model output saved in the CMIP5 archive and therefore can be used to test whether the diffusive approximation of heat and carbon uptake is accurate, to estimate values for *μ* and to test whether heat and carbon uptake are linked in models. For heat removal velocity both *N* and *T*_*o*_ are subject to large internal variability, therefore both variables are filtered with a 7 year moving average for plotting in Fig. 3. Removal velocity is analogous to the ‘piston velocity’ parameter used in diffusive gas exchange problems^19^.

### Heat and carbon ratios from observations

The carbon ratio can be estimated from the change in atmospheric CO_2_ and change in ocean carbon content since the beginning of industrialization. Observation derived estimates of both of these quantities were produced for the fifth assessment report of the IPCC (IPCC AR5)^22^. The estimate for change in atmospheric CO_2_ is 240 ± 10 PgC and for cumulative ocean carbon uptake is 155 ± 30 PgC. To compute an estimate of the carbon ratio we take the IPCC ranges to be the 90% uncertainty range and assume a normal distribution of uncertainty. A simple Monte-Carlo error propagation is performed to estimate the uncertainty on the carbon ratio.

The heat ratio can be estimated from the total radiative forcing and planetary heat uptake. Total radiative forcing was estimated by IPCC AR5^31^ at 2.3 [1.1 to 3.3] W m^−2^. The estimate of planetary heat uptake was taken from ref. 32 and is 0.62 ± 0.43 W m^−2^. The heat ratio is $\frac{L}{N}$, which is equivalent to $\frac{F-N}{N}$. We assume that the uncertainty in *F* and *N* are normally distributed and use Monte-Carlo error propagation to compute the uncertainty bound for the heat ratio.

### Historical emissions comparison

ZD^2^OM emission rates are computed from numerical integration of Equations (13), and (16) forced with the historical CO_2_ concentrations. The historical CO_2_ concentrations are taken from the RCP archive to year 2005^30^ and extended to 2016 using data from Mauna Loa ref. 45. Historical CO_2_ emission estimates are from ref. 46 updated to the present using data from ref. 23. Uncertainty bounds are from Table 6.1 of ref. 22 for decades since the 1980s. Before the 1980s uncertainty bounds for yearly CO_2_ emissions are unavailable.

## Electronic supplementary material

Supplementary material
