# Supplementary material for: The oceanic origin of path-independent carbon budgets
Source: Sci Rep. 2017 Sep 4;7:10373. doi: 10.1038/s41598-017-10557-x (PMC5583191; doi:10.1038/s41598-017-10557-x)
Supplement: Supplementary file 1 — Supplementary material [file 41598_2017_10557_MOESM1_ESM.pdf]

Supplementary material for: The oceanic origin of path-independent carbon budgets

Andrew H. MacDougall

S1 Full derivation of the zero dimensional diffusive ocean model

The zero dimensional diffusive ocean model (ZD^{2}OM) is a simple model of energy and carbon uptake by the ocean that is simple enough to be amenable to analysis. The model is derived from the forcing-response equation (Wigley and Schlesinger, 1985), the Matthews et al. (2009) definition of the Transient Climate Response to Cumulative CO_2 Emissions (TCRE), the ocean carbonate chemistry approximation of Broecker and Peng (1982), and the assumption that the ocean heat and carbon uptake are governed by diffusion.

The Matthews et al. (2009) definition of TCRE is:

$$\Lambda = \frac{T}{E}, \tag{1}$$

where Λ is TCRE, T is the change in global mean temperature, and E is cumulative emissions of CO_2. For the ZD^{2}OM both T and E are subject to approximations that are detailed below.

S1.1 Approximation of temperature evolution

Global mean temperature change (T) is derived from the forcing-response equation (Wigley and Schlesinger, 1985):

$$F = \lambda T + N, \tag{2}$$

where F is the radiative forcing, λ is the climate feedback parameter, and N is planetary heat uptake. We assume that all radiative forcing is from changes in the atmospheric concentration of CO_2 and that all heat uptake goes into the ocean. Therefore we approximate F using the classical approximation of radiative forcing from CO_2 (Myhre et al., 1998):

$$F = R\ln(\frac{C_A}{C_{Ao}}), \tag{3}$$

where R is the radiative forcing from an e-fold increase in CO_2, C_A is the atmospheric CO_2 content, and C_{Ao} is the original atmospheric CO_2 content. N is approximated at a function of temperature:

$$N = \kappa(t)T, \tag{4}$$

Where κ is a time-evolving parameter that relates ocean heat uptake to change in global temperature and is equivalent to the classical ocean heat uptake efficiency (Raper et al., 2002). Substituting and rearranging these equations we find the relationship for temperature:

$$T = R\left(\frac{\ln(\frac{C_A}{C_{Ao}})}{\lambda + \kappa(t)}\right). \tag{5}$$

S1.1.1 The evolution of κ

As a mathematical convenience κ is often treated as a constant (e.g. Raper et al., 2002, Gregory and Forster, 2008). However, it is well understood that this parameter will diminishes toward zero as the climate system comes into equilibrium with the altered radiative forcing and ocean heat uptake ceases (e.g. Wigley and Schlesinger, 1985). Here we account for this evolution by treating ocean heat uptake as diffusion into a half-space. This is a special solution to the general heat equation:

$$\frac{\partial S}{\partial t} = -\alpha\frac{\partial^2 S}{\partial z^2}, \tag{6}$$

where S is a state variable (e.g. temperature), t is time, α is diffusivity, and z is a spatial dimension (Carslaw and Jaeger, 1986). When Equation 6 is solved for a linear increase in surface forcing into a half-space and assuming a uniform initial condition, we find:

$$\frac{S(z,t) - S_i}{rt} = 1 - \text{erf}(\eta) + 2\eta^2 - 2\eta^2\,\text{erf}(\eta) - \frac{2}{\sqrt{\pi}}\eta e^{-\eta^2}, \tag{7}$$

where r is the rate of change in the state variable (assumed to be constant), and η is the modified Fourier modulus $\eta = \frac{z}{\sqrt{4\alpha t}}$ (page 196 of VanSant (1980)). To calculate the surface flux we wish to compute the depth-gradient of the state variable at $z = 0$. Thus we take the partial derivative of the Equation 7 with respect to z, giving us:

$$\frac{\partial S}{\partial z} = rt\left(-\frac{z\,\text{erf}(\eta)}{\alpha t} + \frac{z^2 e^{-\eta^2}}{2\alpha\pi t\sqrt{\alpha t}} - \frac{z^2 e^{-\eta^2}}{2\sqrt{\pi}\alpha t\sqrt{\alpha t}} - \frac{e^{-\eta^2}}{\pi\sqrt{\alpha t}} - \frac{e^{-\eta^2}}{\sqrt{\pi\alpha t}} + \frac{z}{\alpha t}\right). \tag{8}$$

Surface flux is the gradient at $z = 0$ multiplied by the conductivity of the substance αG, and with the direction reversed such into the surface is positive. Therefore the above relationship simplifies to:

$$Q = \frac{G\sqrt{\alpha}(1 + \sqrt{\pi})(S_s(t) - S_i)}{\pi\sqrt{t}}, \tag{9}$$

where Q is flux. Applied to ocean heat flux this equation becomes:

$$\frac{N}{f_o} = \frac{\rho C_p \sqrt{\alpha}\,(1+\sqrt{\pi})\,T_o}{\pi\sqrt{t}}, \tag{10}$$

where f_o is the fraction of the planet covered by ocean, ρC_p is the volumetric heat capacity of water, T_o is the global mean ocean temperature anomaly. Given that $\kappa \equiv \frac{N}{T}$ this gives a relationship for κ of:

$$\kappa(t) = \frac{f_o \rho C_p \epsilon \sqrt{\alpha}\,(1+\sqrt{\pi})}{\pi\sqrt{t}}, \tag{11}$$

where ϵ is the ratio of sea surface temperature change to global temperature change, taken to be a constant. Table S1 shows ϵ values computed for the decades since the 1950s calculated from NOAA global temperature data (Zhang et al., 2017). The decadal average ratio of ocean surface temperature to global temperature change has been relatively stable since the anthropogenic warming signal emerged from climate variability, with values between 0.79 to 0.85. Similarly analysis of climate model output from the CMIP5 archive and the UVic ESCM suggest that the simulated decadal ϵ values are relatively constant in time (Figure S1).

Table S1: Global and ocean temperature anomalies for decades since from the 1950 CE. Ratio of sea surface temperature anomaly to global temperature anomaly (ϵ) is relatively consistent through time. Anomalies are relative to the 1880 to 1910 normal. Data is from NOAA Merged Land Ocean Global Surface Temperature Analysis (Zhang et al., 2017).

Decade	Ocean Temperature Anomaly (°C)	Global Temperature Anomaly (°C)	Ratio ϵ
1950s	0.18	0.21	0.84
1960s	0.20	0.25	0.82
1970s	0.24	0.29	0.85
1980s	0.42	0.50	0.85
1990s	0.52	0.64	0.82
2000s	0.65	0.82	0.79
2010s	0.78	0.97	0.80

To reduce the number of constants we define:

$$\mu = \frac{\pi^2}{\alpha\,(1+\sqrt{\pi})^2}, \tag{12}$$

and:

$$\gamma = f_o \rho C_p \epsilon, \tag{13}$$

to give:

$$\kappa(t) = \frac{\gamma}{\sqrt{\mu t}}. \tag{14}$$

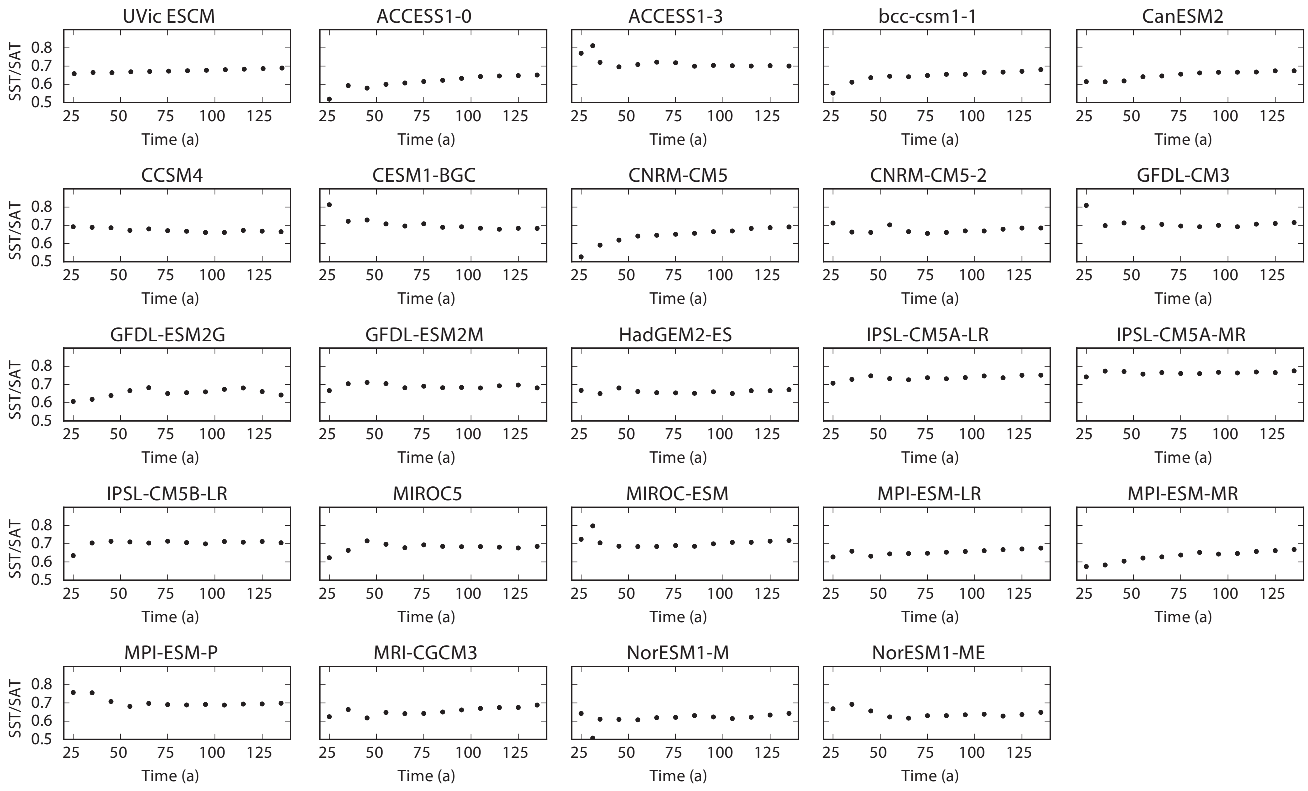

Figure S1: Ratio of the change in Sea Surface Temperature (SST) to change in Surface Air Temperature (SAT) for 22 CMIP5 models and the UVic ESCM. Results are shown for the idealized 1% experiment. Decadal averages are used for both quantities to average out internal variability.

Combining this approximations for κ with Equation 5 we get:

$$T = \frac{R}{\lambda}\left(\frac{\ln(\frac{C_A}{C_{Ao}})}{1+\frac{\gamma}{\sqrt{\mu\lambda^2 t}}}\right). \tag{15}$$

The approximation for κ derived in Equation 14 is fitted to Earth system model (ESM) output from CMIP5 and the UVic ESCM and shown in Figure S2. Values of μ and α are shown in Table S2. The figure shows that the diffusive approximation works well for most Earth system models after the first 20 or so years of the simulation.

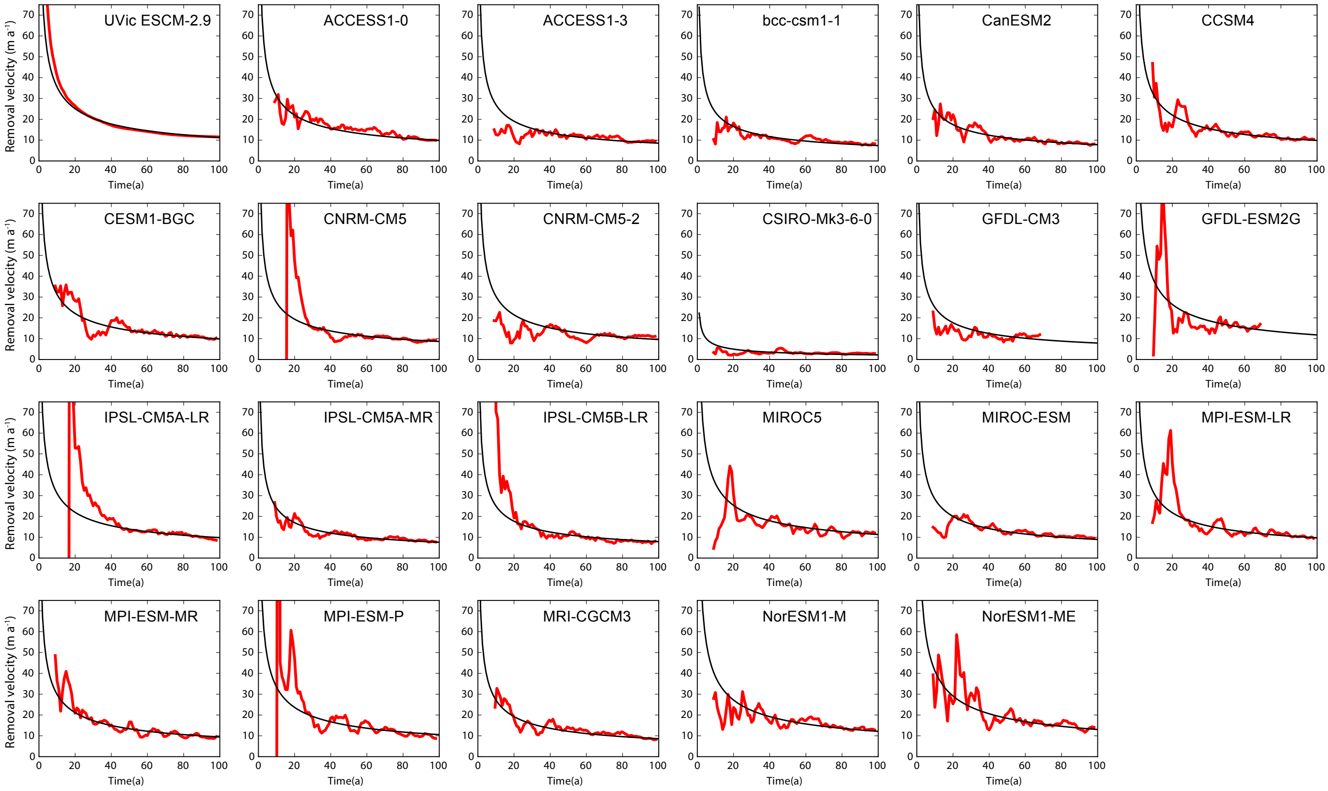

Figure S2: Fit between ocean heat removal velocity $\frac{\kappa}{f_o \rho C_p \epsilon}$ and ocean diffusive approximation of removal velocity $\frac{1}{\sqrt{\mu t}}$ for UVic ESCM and all CMIP5 models that saved all necessary output. In all cases the 1% experiment is examined. Red lines are climate model output and black line is the fit to the diffusive approximation.

Table S2: Values of μ, α (ocean diffusivity), and correlation coefficients for fits between for 22 CMIP5 models the UVic ESCM, and diffusive approximation of ocean heat removal velocity.

Model	μ ($\mathrm{a\,m^{-2}}$)	α ($\mathrm{cm^2\ s^{-1}}$)	Correlation coefficient
UVic ESCM 2.9F	8.0e-5	5.09	0.99
ACCESS1-0	10.3e-5	3.95	0.97
ACCESS1-3	13.7e-5	2.97	0.86
bcc-csm1-1	18.3e-5	2.22	0.87
CanESM2	16.4e-5	2.48	0.87
CCSM4	10.3e-5	3.95	0.90
CESM1-BGC	10.2e-5	3.98	0.69
CNRM-CM5	13.3e-5	3.06	0.88
CNRM-CM5-2	10.9e-5	3.73	0.72
CSIRO-Mk3-6-0	201.4e-5	0.20	0.65
GFDL-CM3	16.0e-5	2.54	0.73
GFDL-ESM2G	7.2e-5	5.65	0.78
IPSL-CM5A-LR	10.3e-5	3.95	0.95
IPSL-CM5A-MR	17.3e-5	2.35	0.88
IPSL-CM5B-LR	16.0e-5	2.54	0.94
MIROC5	7.8e-5	5.22	0.88
MIROC-ESM	12.5e-5	3.26	0.96
MPI-ESM-LR	10.6e-5	3.84	0.89
MPI-ESM-MR	11.2e-5	3.63	0.94
MPI-ESM-P	8.9e-5	4.57	0.86
MRI-CGCM3	13.5e-5	3.01	0.89
NorESM1-M	6.8e-5	5.98	0.86
NorESM1-ME	5.9e-5	6.90	0.85

S1.2 Approximation of cumulative emissions

Cumulative CO_2 emissions are the sum of the carbon added to the atmosphere, ocean, and terrestrial biosphere from anthropogenic sources. For the ZD^2OM we assume that the uptake by land is a constant fraction of anthropogenic emissions (l) and that the change in the atmospheric carbon pool is known. Therefore the evolution of emissions is given by the equation:

$$E = \frac{1}{1-l}\left(C_A - C_{Ao} + \int_o^t q_o \, dt\right), \tag{16}$$

where E is cumulative emissions of CO_2 and q_o is the flux of carbon into the ocean. We approximate q_o using a similar approximation as that used for κ:

$$q_o = B_o \frac{D_s - D_{so}}{\sqrt{\mu t}}, \tag{17}$$

where D_s is the surface Dissolved Inorganic Carbon (DIC) of the ocean, D_{so} is the original surface DIC of the ocean and B_o is a conversion factor equal to:

$$B_o = \frac{A_o M}{1 \times 10^{15}}, \tag{18}$$

where A_o is the area of the ocean, M is the molar mass of carbon, and 1×10^{15} is the conversion factor between g and Pg. For Equation 17 we have assumed that the forcing at the surface of the ocean will be linear and therefore we can use the same solution to the heat equation that we used for κ. We show in section S2 that this assumption is justified.

Equation 17 can be re-arranged to be fitted to Earth system model output from CMIP5 and the UVic ESCM, such that $\frac{1}{\sqrt{\mu t}} = \frac{q_o}{B_o(D_s - D_{so})}$.The fit between this function and ESM output is shown in Figure S3 and the value of μ and α derived from this fitting are shown in Table S3. The figure shows good fits between the approximation and the model output for most of the models, with the exception of CNRM-CM5. In most models the fit is best for the mid part of the simulation (roughly years 40 to 80) and poorer for the first 20 years of the simulation.

Substituting Equation 17 into Equation 16 we find:

$$E = \frac{1}{1-l}\left(C_A - C_{Ao} + \int_o^t B_o \frac{D_s - D_{so}}{\sqrt{\mu t}} \, dt\right), \tag{19}$$

an equation that can be integrated to find $E(t)$.

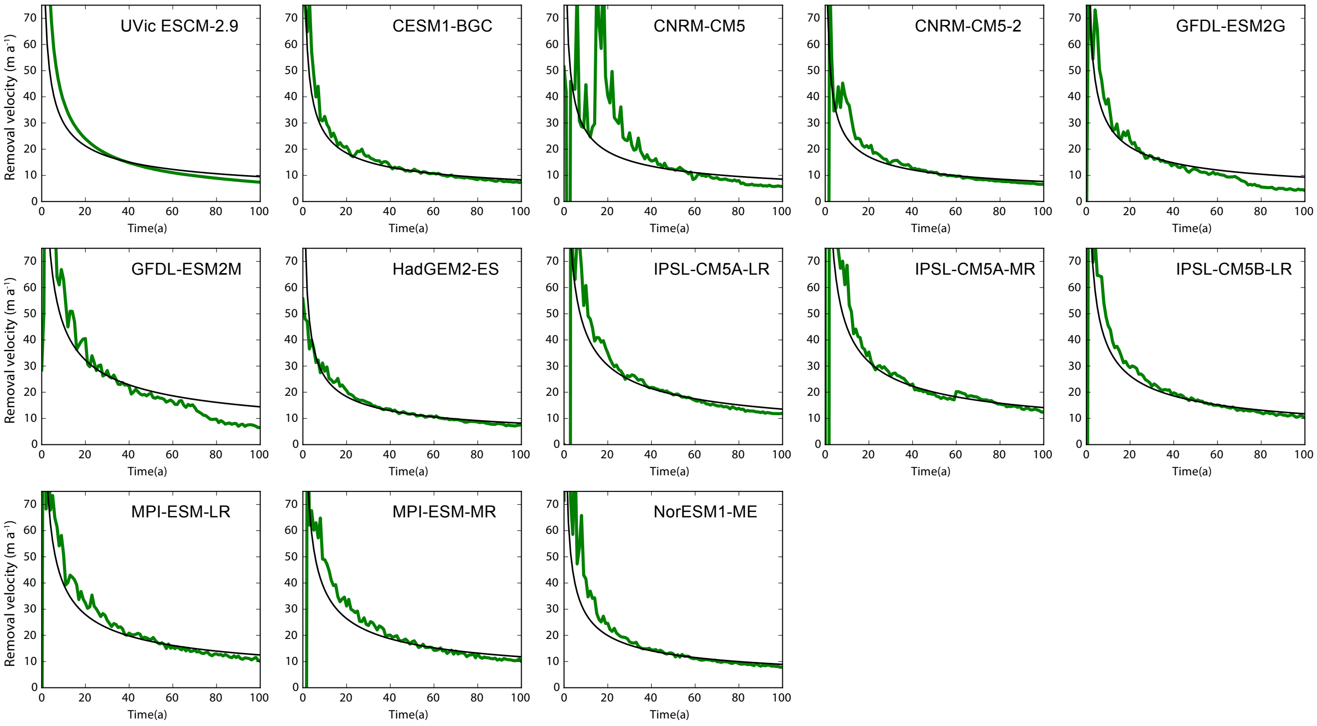

Figure S3: Fit between ocean carbon removal velocity $\frac{q_o}{B_o(D_s - D_{so})}$ and ocean diffusive approximation of q_o (Equation 17) for UVic ESCM and all CMIP5 models that saved all necessary output. Green lines are climate model output and black line is the fit. Note that GFDL stabilizes atmospheric CO_2 concentration at double the pre-industrial concentration in year 70 of the 1% experiment, while the other modelling groups continue the 1% experiment until $4 \times CO_2$ was reached.

Table S3: Values of μ, α (ocean diffusivity), and correlation coefficients for fits between for 12 CMIP5 models the UVic ESCM, and diffusive approximation of ocean carbon removal velocity.

Model	μ ($\mathrm{a\,m^{-2}}$)	α ($\mathrm{cm^2\ s^{-1}}$)	Correlation coefficient
UVic ESCM 2.9F	11.1e-5	3.67	0.998
CESM1-BGC	14.6e-5	2.78	0.99
CNRM-CM5	13.7e-5	2.97	0.80
CNRM-CM5-2	17.1e-5	2.38	0.99
GFDL-ESM2G	11.5e-5	3.54	0.98
GFDL-ESM2M	4.8e-5	8.48	0.98
HadGEM2-ES	14.9e-5	2.73	0.997
IPSL-CM5A-LR	5.4e-5	7.53	0.99
IPSL-CM5A-MR	5.0e-5	8.14	0.98
IPSL-CM5B-LR	7.2e-5	5.65	0.998
MPI-ESM-LR	6.4e-5	6.36	0.99
MPI-ESM-MR	7.2e-5	5.65	0.997
NorESM1-ME	12.6e-5	3.22	0.99

S1.3 Complete zero dimensional diffusive ocean model

Substituting our equations for T and E (Equations 15 and 16) into Equation 1 we find:

$$\Lambda = \frac{R(1-l)}{\lambda}\left(\frac{\ln(\frac{C_A}{C_{Ao}})}{1+\frac{\gamma}{\sqrt{\mu\lambda^2 t}}}\right)\left(\frac{1}{C_A - C_{Ao} + \int_o^t B_o \frac{D_s - D_{so}}{\sqrt{\mu t}}\, dt}\right). \tag{20}$$

To further evaluate this equation we must link the change in atmospheric CO_2 to the change in the surface concentration of DIC (C_A with D_s). Therefore we incorporate an approximation of ocean carbonate chemistry into our model.

S2 Carbonate Chemistry

S2.1 Basic relationships

When CO_2 dissolves in water it reacts with the water to form carbonic acid, H_2CO_3 which in turn dissociates into bicarbonate and carbonate ions. The reactions are written:

$$\mathrm{CO_2(aq)} + \mathrm{H_2O} \rightleftharpoons \mathrm{H_2CO_3}, \tag{21}$$

$$\mathrm{H_2CO_3} \rightleftharpoons \mathrm{HCO_3}^- + \mathrm{H}^+, \tag{22}$$

$$\mathrm{HCO_3}^- + \mathrm{H_2O} \rightleftharpoons \mathrm{CO_3}^{2-} + \mathrm{H}^+. \tag{23}$$

These reactions proceed to equilibrium within minutes and therefore have simple equilibrium constants:

$$K_1 = \frac{[\mathrm{HCO_3}^-][\mathrm{H}^+]}{[\mathrm{CO_2}^*]}, \tag{24}$$

$$K_2 = \frac{[\mathrm{CO_3}^{2-}][\mathrm{H}^+]}{[\mathrm{HCO_3}^-]}, \tag{25}$$

where $[\mathrm{CO_2}^*]$ is the combined concentration of $\mathrm{CO_2}_{\mathrm{aq}}$ and H_2CO_3. Both equilibrium constants are weak functions of temperature and salinity (Broecker and Peng, 1982).

CO_2 will dissolve in ocean-water if the partial pressure of CO_2 is higher in the atmosphere than in ocean-water. The partial pressure of dissolved CO_2 is determined by:

$$pCO_2 = \frac{[\mathrm{CO_2}^*]}{K_o}, \tag{26}$$

where K_o is the solubility of CO_2, a function of temperature and salinity.

The inorganic carbon contented of the ocean is the sum of the species of dissolved inorganic carbon (DIC):

$$D = [{CO_2}^*] + [{HCO_3}^-] + [{CO_3}^{2-}], \tag{27}$$

where D is DIC. DIC constitutes the vast majority of carbon stored in the ocean (e.g. Ciais et al., 2013) and therefore the evolution of DIC as carbon is released to the atmosphere is the principle driver of ocean carbon uptake.

To solve the carbonate system of equations for a specific component an additional constraint is needed in the form of ocean alkalinity. Alkalinity is near-constant on timescales shorter than terrestrial weathering and dissolution of ocean sediments (e.g. Archer, 1996). The full equation for alkalinity is:

$$A_t = [{HCO_3}^-] + 2\,[{CO_3}^{2-}] + [{B(OH)_4}^-] + [OH^-] + [{HPO_4}^{2-}] + 2\,[{PO_4}^{3-}] + [{SiO(OH)_3}^-] - [H^+] - [{HSO_4}^-], \tag{28}$$

(e.g. Follows et al., 2006).

S2.2 Solving for ocean carbon uptake

For our purposes we wish to solve the carbonate system analytically to derive a relationship between atmospheric CO_2 concentration and ocean surface DIC. To make this possible we will make three simplifying assumptions: 1) we will ignore the temperature and salinity dependence of K_1, K_2 and K_o. 2) We will approximate alkalinity as carbonate alkalinity following Broecker and Peng (1982), and 3) we will assume that at the surface the partial pressure of CO_2 in the ocean is equal to the partial pressure of CO_2 in the atmosphere.

For the purposes of analysis of the potential of ocean system to take up carbon Broecker and Peng (1982) approximated ocean alkalinity as carbonate alkalinity:

$$A_c = [{HCO_3}^-] + 2\,[{CO_3}^{2-}]. \tag{29}$$

This approximation allows for the carbonate system of equations to be solved analytically but is not accurate enough to be used in ESMs. Follows et al. (2006) conducted an error analysis of the approximation and found it to be accurate to within 1 ppmv pCO_2. The approximation forms the basis of some numerical methods of solving the full carbonate system (e.g. Follows et al., 2006).

Using these simplifications the surface ocean concentration of DIC is given by:

$$D_s = [{CO_2}^*] + \frac{1}{2}\sqrt{\frac{K_1^2}{16K_2^2}[{CO_2}^*]^2 + \frac{K_1}{2K_2}[{CO_2}^*]A_c} - \frac{K_1}{8K_2}[{CO_2}^*] + \frac{A_c}{2}, \tag{30}$$

where D_s is the surface DIC concentration. Applying equation 26 and our assumption that at the surface ocean pCO_2 is in equilibrium with atmospheric CO_2 the equation becomes:

$$D_s = \frac{1}{2}\sqrt{\frac{K_1^2 K_o^2}{16 K_2^2 M_r^2} C_A^2 + \frac{K_1 K_o}{2 K_2 M_r} C_A A_c} + \left(\frac{K_o}{M_r} - \frac{K_1 K_o}{8 K_2 M_r}\right) C_A + \frac{A_c}{2}, \tag{31}$$

where M_r is the mixing ratio, the conversion factor between the mass of CO_2 in the atmosphere and the partial pressure of CO_2 in the atmosphere.

The above equation can be substituted into the equation for TCRE (Equation 20) to solve to the evolution of TCRE in time. However the integral in the equation, $\int_o^t B_o \frac{D_s - D_{so}}{\sqrt{\mu t}} dt$, cannot be solved analytically so the system must be solved using numerical integration. Although such an integration would be more accurate its does not further our goal of determining the source of the rate independence of TCRE, as numerical integration simply becomes another 'black box'. Therefore we must approximate Equation 31 to complete our analysis.

S2.3 Approximation of surface DIC evolution

To find an approximate analytical solution for the integral in Equation 20 we need an approximation of D_s. The simplest choice is a Taylor-series approximation of the function. Such an approximation is shown in Figure S4. From the figure it is clear that Taylor-series approximation is accurate only over a very small range of atmospheric CO_2 concentrations. Equation 31 is a square-root function and therefore transforming the function into log-space and computing a Taylor series in such a space should yield a more accurate approximation. Figure S4 shows that such is the case and that the log-space approximation of D_s is reasonably accurate (below 0.5% error) between 180 to 600 ppm of atmospheric CO_2. The log-space Taylor series approximation gives:

$$D_s \approx \left(C_{Ao}\left(\frac{K_o}{M_r} - \frac{K_1 K_o}{8 K_2 M_r}\right) + \frac{\frac{K_1^2 K_o^2}{8 K_2^2 M_r^2} C_{Ao}^2 + \frac{K_1 K_o}{2 K_2 M_r} C_{Ao} A_c}{4\sqrt{\frac{K_1^2 K_o^2}{16 K_2^2 M_r^2} C_{Ao}^2 + \frac{K_1 K_o}{2 K_2 M_r} C_{Ao} A_c}} \right) \ln\left(\frac{C_A}{C_{Ao}}\right) + D_{so} \tag{32}$$

For simplicity we define a constant Γ to represent the factor in front of the logarithm:

$$\Gamma = \left(C_{Ao}\left(\frac{K_o}{M_r} - \frac{K_1 K_o}{8 K_2 M_r}\right) + \frac{\frac{K_1^2 K_o^2}{8 K_2^2 M_r^2} C_{Ao}^2 + \frac{K_1 K_o}{2 K_2 M_r} C_{Ao} A_c}{4\sqrt{\frac{K_1^2 K_o^2}{16 K_2^2 M_r^2} C_{Ao}^2 + \frac{K_1 K_o}{2 K_2 M_r} C_{Ao} A_c}} \right). \tag{33}$$

This factor is equivalent to the increase in ocean surface DIC given an e-fold increase in atmospheric CO_2 concentration. Substituting this relationship into the equation for ocean carbon flux (Equation 17) we find:

$$q_o \approx \frac{B_o \Gamma \ln(\frac{C_A}{C_{Ao}})}{\sqrt{\mu t}}, \tag{34}$$

and the relationship for TCRE becomes:

$$\Lambda \approx \frac{R(1-l)}{\lambda}\left(\frac{\ln(\frac{C_A}{C_{Ao}})}{1+\frac{\gamma}{\sqrt{\mu\lambda^2 t}}}\right)\left(\frac{1}{C_A - C_{Ao} + B_o\Gamma\int_0^t \frac{\ln(\frac{C_A}{C_{Ao}})}{\sqrt{\mu t}}\,dt}\right). \tag{35}$$

Under certain special conditions Equation 35 is amenable to analytic solutions.

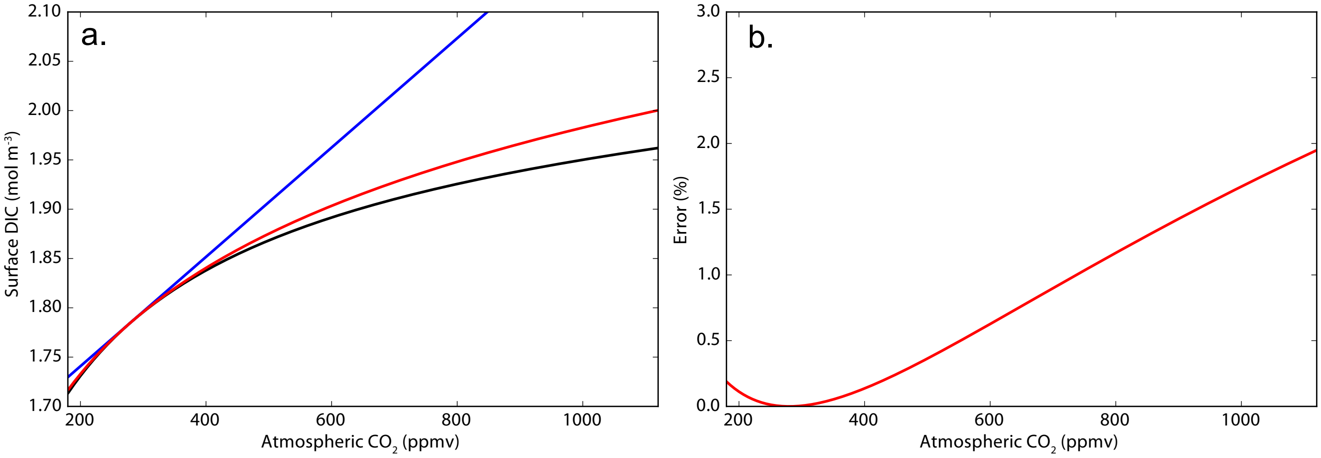

Figure S4: (a)Function for ocean surface dissolved inorganic carbon (black line), log-space Taylor-series approximation (red line), and Euclidian-space Taylor series approximation (blue line). (b) Error between logarithmic approximation and actual function. Functions are computed using standard parameter values found in Table S4.

S3 Solutions to the zero dimensional diffusive ocean model

The approximate form of the ZD^{2}OM shown in Equation 35 can be solved analytically for certain CO_2 concentration pathways. Two pathways relevant to the climate problem are a linear increase in atmospheric CO_2 concentration:

$$C_A = bt + C_{Ao}, \tag{36}$$

and an exponential increase in atmospheric CO_2 concentration:

$$C_A = C_{Ao}e^{\beta t}, \tag{37}$$

where b is the rate of change in atmospheric CO_2 content ($\mathrm{Pg\,a^{-1}}$) and β is the growth rate of atmospheric CO_2 content ($\%\,\mathrm{a}^{-1}$). Solutions for these two pathways are described in the following subsections.

S3.1 Solution for a linear increase in atmospheric CO_2

For the case of a linear increase in atmospheric CO_2 concentration the integral from Equation 34 becomes:

$$B_o \int_0^t \frac{\Gamma \ln(\frac{bt+C_{Ao}}{C_{A}o})}{\sqrt{\mu t}}\, dt = \frac{-4B_o b\Gamma}{\mu^2}\left(\frac{\mu\sqrt{\mu t}}{b} - \frac{C_{Ao}\mu^2}{b\sqrt{b\mu C_{Ao}}}\arctan\left(\frac{b\sqrt{\mu t}}{\sqrt{b\mu C_{Ao}}}\right)\right) + \frac{2B_o\Gamma}{\mu}\ln\left(\frac{bt+C_{Ao}}{C_{Ao}}\right)\sqrt{\mu t}. \tag{38}$$

Substituting $t = \frac{C_a - C_{ao}}{b}$ into the above relationship and Equation 35 we get TCRE as a function of C_A:

$$\Lambda \approx \frac{R(1-l)}{\lambda}\left(\frac{\ln(\frac{C_A}{C_{Ao}})}{1+\frac{\gamma\sqrt{b}}{\sqrt{\mu\lambda^2(C_A - C_{Ao})}}}\right)\times \tag{39}$$

$$\left(\frac{1}{C_A - C_{Ao} - \frac{4B_o\Gamma}{\sqrt{b\mu}}\left(\sqrt{C_A - C_{Ao}} - \sqrt{C_{Ao}}\arctan\left(\frac{\sqrt{C_A - C_{Ao}}}{\sqrt{C_{Ao}}}\right)\right) + \frac{2B_o\Gamma}{\sqrt{b\mu}}\ln\left(\frac{C_A}{C_{Ao}}\right)\sqrt{C_A - C_{Ao}}}\right). \tag{40}$$

(41)

S3.2 Solution for a exponential increase in atmospheric CO_2

For an exponential increase in the atmospheric CO_2 concentration the integral from Equation 34 becomes:

$$B_o\Gamma \int_0^t \frac{\sqrt{t}}{\sqrt{\mu}}\, dt = \frac{2B_o\Gamma\beta}{3\sqrt{\mu}} t^{\frac{3}{2}}. \tag{42}$$

Substituting this relationship into Equation 35 we find:

$$\Lambda \approx \frac{R(1-l)}{\lambda}\left(\frac{\ln(\frac{C_A}{C_{Ao}})}{1+\frac{\gamma\sqrt{\beta}}{\sqrt{\mu\lambda^2\ln(\frac{C_A}{C_{Ao}})}}}\right)\left(\frac{1}{C_A - C_{Ao} + \frac{2B_o\Gamma\ln(\frac{C_A}{C_A o})^{\frac{3}{2}}}{3\sqrt{\mu\beta}}}\right). \tag{43}$$

S4 Value of parameters and constants

Table S4: Value of parameters and constants used in the Main Text and Supplementary Information. Citations are given to source of values, except for derived parameters or common knowledge. Value for the climate feedback parameter is that consistent with climate sensitivity of 3.0°C for doubling of atmospheric CO_2 concentration.

Constant	Description	Units	Value	Source
A_c	Carbonate alkalinity: $[HCO_3{}^-] + 2\,[CO_3{}^{2-}]$	$mol\,m^{-3}$	2.015	Broecker and Peng (1982)
B_o	Unit conversion constant (carbon)	$m^2\,Pg\,mol^{-1}$	4.32	–
C_{Ao}	Pre-Industrial atm. CO_2	PgC	596	Ciais et al. (2013)
D_{so}	Pre-Industrial ocean suface DIC	$mol\,m^{-3}$	2.0	Broecker and Peng (1982)
f_o	Planetary ocean fraction	–	0.7058	–
K_1	Equilibrium constant: $[HCO_3{}^-][H^+]/[CO_2{}^*]$	$mol\,m^{-3}$	$10^{-2.86}$	Broecker and Peng (1982)
K_2	Equilibrium constant: $[CO_3{}^{2-}][H^+]/[HCO_3{}^-]$	$mol\,m^{-3}$	$10^{-5.95}$	Broecker and Peng (1982)
K_o	Solubility of CO_2	$ppm^{-1}\,m^{-3}\,mol$	$10^{-1.54}$	Broecker and Peng (1982)
l	Land-borne fraction of carbon	–	0.25	Ciais et al. (2013)
M_r	CO_2 atmospheric mixing ratio	$Pg\,ppm^{-1}$	2.13	
R	e-fold radiative forcing from CO_2	$W\,m^{-2}$	5.35	Myhre et al. (1998)
α	Ocean diffusivity	$cm^2 s^{-1}$	1.0	Munk (1966)
Γ	Surface DIC change from e-fold change in atm. CO_2	$mol\,m^{-3}$	0.155	–
ϵ	Ratio of global to ocean T change	–	0.67	Stocker et al. (2013)
λ	Climate feedback parameter	$W\,m^{-1}K^{-1}$	1.24	Collins et al. (2013)
ρC_p	Heat capacity of water	$J\,m^{-3}K^{-1}$	4.18×10^6	–
τ	Unit conversion constant (heat)	$s\,a^{-1}$	3.171×10^{-8}	–

S5 Ocean surface DIC model comparison

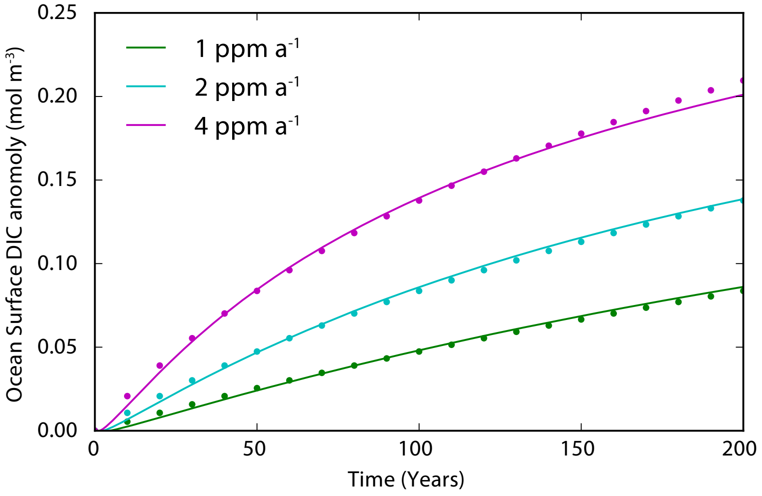

Figure S5: Comparison of global ocean surface Dissolved Inorganic Carbon (DIC) anomaly in the UVic ESCM (solid lines) and the same quantity calculated from Equation 32 (dotted lines). Simulations are from the linear increase in atmospheric CO_2 concentration model experiments.

S6 Proof of rate term cancelation

We have two functions, one is a rational square-root function $y_1 = Ax^{-\frac{1}{2}}$ and the other is a square-root function $y_2 = Bx^{\frac{1}{2}}$. These two functions have the same value at $x = \frac{A}{B}$. We wish to prove that where these two functions are of equal value their derivatives are of equal magnitude but of opposite sign. That is:

$$\frac{dy_2}{dx} = -\frac{dy_1}{dx}, \tag{44}$$

where $x = \frac{A}{B}$. Taking the derivative of y_1 and y_2 we find:

$$\frac{dy_1}{dx} = -\frac{1}{2}Ax^{-\frac{3}{2}}, \tag{45}$$

and:

$$\frac{dy_2}{dx} = \frac{1}{2}Bx^{-\frac{1}{2}}. \tag{46}$$

Thus if we substitute x into the Left Hand Side (LHS) of Equation 44, which is Equation 45, we find:

$$LHS = \frac{1}{2}BA^{-\frac{1}{2}}B^{\frac{1}{2}}, \tag{47}$$

which reduces to:

$$LHS = \frac{1}{2}A^{-\frac{1}{2}}B^{\frac{3}{2}}. \tag{48}$$

Following a similar procedure and substituting x into the Right Hand Side (RHS) of Equation 44, which is Equation 46, we find:

$$RHS = \frac{1}{2}AA^{-\frac{3}{2}}B^{\frac{3}{2}}, \tag{49}$$

which reduces to:

$$RHS = \frac{1}{2}A^{-\frac{1}{2}}B^{\frac{3}{2}}. \tag{50}$$

We can see that:

$$LHS = RHS \tag{51}$$

quod erat demonstrandum.

Therefore where one term is a square-root function and the other is a rational square-root function their derivatives will cancel exactly where the two terms are of equal value.

References

Archer, D., 1996: A data-driven model of the global calcite lysocline. *Global Biogeochemical Cycles*, **10 (3)**, 511–526.

Broecker, W. and T. Peng, 1982: *Tracers in the Sea*. Eldigio Press, 690 pp.

Carslaw, H. S. and J. C. Jaeger, 1986: *Conduction of heat in solids*. Oxford University Press, 510 pp.

Ciais, P., et al., 2013: Carbon and other biogeochemical cycles. *Working Group I Contribution to the Intergovernmental Panel on Climate Change Fifth Assessment Report Climate Change 2013: The Physical Science Basis*, T. F. Stocker, D. Qin, G.-K. Plattner, M. Tignor, S. K. Allen, J. Boschung, A. Nauels, Y. Xia, V. Bex, and P. Midgley, Eds., Cambridge University Press.

Collins, M., et al., 2013: Long-term climate change: Projections, commitments and irreversibility. *Working Group I Contribution to the Intergovernmental Panel on Climate Change Fifth Assessment Report Climate Change 2013: The Physical Science Basis*, Cambridge University Press.

Follows, M. J., T. Ito, and S. Dutkiewicz, 2006: On the solution of the carbonate chemistry system in ocean biogeochemistry models. *Ocean Modelling*, **12 (3)**, 290–301.

Gregory, J. and P. Forster, 2008: Transient climate response estimated from radiative forcing and observed temperature change. *Journal of Geophysical Research:*, **113 (D23)**.

Matthews, H. D., N. P. Gillett, P. A. Stott, and K. Zickfeld, 2009: The proportionality of global warming to cumulative carbon emissions. *Nature*, **459**, 829–832, doi:10.1038/nature08047.

Munk, W., 1966: Abyssal recipes. *Deep Sea Research*, **13**, 707–730.

Myhre, G., E. J. Highwood, K. P. Shine, and F. Stordal, 1998: New estimates of radiative forcing due to well mixed greenhouse gases. *Geophysical research letters*, **25 (14)**, 2715–2718.

Raper, S. C. B., J. M. Gregory, and R. J. Stouffer, 2002: The role of climate sensitivity and ocean heat uptake on aogcm transient temperature response. *Journal of Climate*, **15 (1)**, 124–130.

Stocker, T., et al., 2013: Technical summary. *Working Group I Contribution to the Intergovernmental Panel on Climate Change Fifth Assessment Report Climate Change 2013: The Physical Science Basis*, T. F. Stocker, D. Qin, G.-K. Plattner, M. Tignor, S. K. Allen, J. Boschung, A. Nauels, Y. Xia, V. Bex, and P. Midgley, Eds., Cambridge University Press.

VanSant, J. H., 1980: *Conduction heat transfer solutions*. Lawrence Livermore National Laboratory, 562 pp.

Wigley, T. M. and M. E. Schlesinger, 1985: Analytical solution for the effect of increasing CO_2 on global mean temperature. *Nature*, **315**, 649–652.

Zhang, H.-M., B. Huang, T. Smith, and R. Ray, 2017: NOAA merged land ocean global surface temperature analysis. National Oceanic and Atmospheric Administration, USA, URL https://www.ncdc.noaa.gov/data-access/marineocean-data/noaa-global-surface-temperature-noaaglobaltemp, URL https://www.ncdc.noaa.gov/data-access/marineocean-data/noaa-global-surface-temperature-noaaglobaltemp
